# Integrated genetic and functional analyses reveal the pathogenic effects of a novel *STXBP3* variant in very-early-onset inflammatory bowel disease with immunodeficiency

**DOI:** 10.3389/fimmu.2026.1871633

**Published:** 2026-10-05

**Authors:** Chun Pan, Ping Zhang, Wenjuan Tang, Shengnan Wang, Zhiheng Huang, Xiaolan Lu, Yingying Meng, Haijiao Xia, Lin Wang, Ying Huang

**Affiliations:** 1 Department of Gastroenterology, Children’s Hospital of Fudan University, National Children’s Medical Center, Shanghai, China; 2 Center of Molecular Medicine, Pediatrics Research Institute, Children’s Hospital of Fudan University, National Children’s Medical Center, Shanghai, China

**Keywords:** genotype-phenotype correlation, primary immunodeficiency, sensorineural hearing loss, STXBP3, VEOIBD

## Abstract

**Background:**

Very early onset inflammatory bowel disease (VEOIBD) is frequently driven by monogenic defects. Pathogenic variants in the syntaxin-binding protein 3 (*STXBP3*) gene have been implicated in VEOIBD; however, the relationship between specific variant types and clinical manifestations remains incompletely understood. This study aimed to identify the genetic cause in a patient with VEOIBD and explore the possible genotype-phenotype correlations of *STXBP3*-related disorders.

**Methods:**

We performed trio-whole-exome sequencing (trio-WES) on a male infant presenting with VEOIBD, sensorineural hearing loss, and immunodeficiency. Functional consequences of the identified variant were investigated using RNA sequencing (RNA-seq), reverse transcription-polymerase chain reaction (RT-PCR), western blotting and co-immunoprecipitation (co-IP) assay. A systematic literature review was conducted to analyze clinical features and inheritance patterns across all reported *STXBP3* cases.

**Results:**

A novel heterozygous splice-site variant in *STXBP3* (c.1022_1029 + 3del) was identified. RNA-seq and RT-PCR confirmed that this variant causes aberrant splicing, leading to the skipping of exon 12 and the production of a truncated protein. Co-IP showed that the aberrant protein reduced the ability to bind STX4. The comprehensive analysis of 13 patients revealed possible genotype-phenotype correlations: splice-site and frameshift variants were observed predominantly follow an autosomal dominant (AD) pattern associated with VEOIBD, sensorineural hearing loss, and immunodeficiency; conversely, missense variants generally follow an autosomal recessive (AR) pattern linked to VEOIBD, and congenital malformations. Given the small cohort, these comparisons were descriptive and were not intended to establish definitive genotype–phenotype associations.

**Conclusions:**

We identified a novel pathogenic *STXBP3* variant and validated its impact on its function. The observed differences between AD and AR cases provided a preliminary, hypothesis-generating framework for understanding STXBP3-related disease, which require validation in larger cohorts.

## 1 Introduction

Inflammatory bowel disease (IBD) encompasses a spectrum of chronic inflammatory disorders affecting the gastrointestinal tract, including ulcerative colitis (UC), Crohn’s disease (CD), and IBD unclassified (IBD-U). Disease onset before 6 years of age is classified as very early-onset IBD (VEOIBD) (1, 2). Although the pathogenesis of IBD is generally considered multifactorial, conditions arising from single-gene variants that follow Mendelian inheritance patterns are distinct entities termed monogenic IBD (3). Recently, the identification of monogenic forms manifesting as IBD or IBD-like intestinal inflammation has expanded significantly (4). Research demonstrates that children diagnosed at a younger age exhibit disease trajectories distinct from those of older children and adults. Compared to late-onset IBD, VEOIBD is typically characterized by greater severity, more extensive disease involvement, a poorer response to conventional immunosuppressive therapies, and a higher prevalence of monogenic etiologies (5). These cases, designated as monogenic IBD (mIBD), represent a critical component of the pediatric disease spectrum. Many forms of monogenic IBD (mIBD) overlap with inborn errors of immunity (IEI), as their underlying mechanisms often involve defects in immune development, host defense, inflammatory regulation, and epithelial barrier homeostasis (6). Accordingly, mIBD frequently presents not only with intestinal inflammation but also with recurrent infections, immune dysregulation, and multisystem extraintestinal manifestations. Thus, mIBD is more appropriately viewed as a gastrointestinal manifestation of systemic immune dysfunction rather than an isolated intestinal inflammatory disease (7).

The syntaxin-binding protein 3 (*STXBP3*) gene, which encodes the protein Munc18c, is a key regulator of membrane trafficking. It facilitates vesicle fusion by binding to its cognate soluble N-ethylmaleimide-sensitive factor attachment protein receptor (SNARE) partner, syntaxin 4 (STX4). In a reconstituted membrane-fusion system, Munc18c was shown to recognize its cognate trans-SNARE complex and promote trans-SNARE zippering at the post-docking stage, thereby accelerating membrane fusion (8). This well-characterized interaction is critical for physiological processes such as insulin-stimulated GLUT4 translocation and platelet secretion (9, 10). The interaction is dynamically regulated by protein kinase C (PKC)-mediated phosphorylation of STXBP3, which promotes its dissociation from STX4 to enable vesicle fusion (11–14). Beyond its fundamental role in cellular secretion, STXBP3 is also implicated in immune regulation. It is expressed in key immune cell lineages, including circulating monocytes, dendritic cells (DCs), B cells, and T cells (15). Additionally, STXBP3 modulates calcium influx and the activation of the transcription factor nuclear factor of activated T cells (NFAT) via the calcineurin pathway (16). Furthermore, STXBP3 dysregulation has been observed in autoimmune conditions such as systemic sclerosis (17).

STXBP3 is indispensable for development, as complete genetic ablation in mice results primarily in embryonic lethality. In humans, heterozygous loss-of-function variants in STXBP3 are associated with very early-onset inflammatory bowel disease (VEOIBD), sensorineural hearing loss, and primary immunodeficiencies characterized by reduced natural killer (NK) cell and CD8^+^ T cell counts (18). These clinical phenotypes are recapitulated in zebrafish models, manifesting as similar pathological changes, including abnormal otolith development and intestinal dysplasia (19). In this study, we report a novel heterozygous *STXBP3* variant identified in a patient presenting with VEOIBD, sensorineural hearing loss, and primary immunodeficiency.

## 2 Materials and methods

### 2.1 Patient evaluation and ethical approval

We conducted a comprehensive evaluation of a 1-month-old patient with recurrent diarrhea and infection at the Children’s Hospital of Fudan University (Shanghai, China), with follow-up until the patient reached 3 years old. Written informed consent for participation was obtained from the patient’s legal guardians. This study was conducted with the approval of the Ethics Committee of the Children’s Hospital of Fudan University (Approval number 2023244).

### 2.2 Whole-exome sequencing

Genomic DNA was isolated from peripheral blood samples obtained from the patient and parents. WES was performed as previously described (20). WES libraries were prepared with the IDTX Genomes2 and sequenced on the Illumina NovaSeq6000 platform (Illumina, San Diego, CA, USA). The mean sequencing depth across the target regions was ≥90×, with 96% of the target regions covered at a depth of >20×. Base calling was performed for all sequenced fragments. Sequence reads were aligned to the UCSC hg19 human reference genome using Sentieon BWA (v0.5.9-r16). In addition, variants were annotated using Variant Effect Predictor (Ensembl release 73) and ANNOVAR.

Variant interpretation incorporated evidence from disease-related databases, including ClinVar and HGMD, as well as population frequency databases, including the 1000 Genomes Project, gnomAD, and ExAC, in order to exclude common variants in the general population. Candidate variants were prioritized according to phenotype relevance and predicted functional impact. The pathogenicity of candidate variants was classified according to the American College of Medical Genetics and Genomics (ACMG) guidelines, and sequence variants were described following the Human Genome Variation Society (HGVS) nomenclature. Phenotype-driven prioritization of candidate genes was performed using Human Phenotype Ontology (HPO) and/or Online Mendelian Inheritance in Man (OMIM) terms based on the patient’s clinical manifestations.

The initial trio-WES analysis was performed using phenotype-driven prioritization based on the terms Diarrhea (HP:0002014), Enterocolitis (HP:0004387) and Hypogammaglobulinemia (HP:0004313). The original WES data were reanalyzed later using an expanded candidate-gene range and broader consideration of the patient’s combined gastrointestinal, immunological, and auditory phenotypes. Bioinformatic processing, variant filtering, and annotation were performed using a previously reported pipeline (21).

### 2.3 Structural modeling

Structural modeling was performed using AlphaFold 3 to predict the interaction between STXBP3 and STX4 (22). The predicted models were visualized and analyzed in PyMOL.

### 2.4 Human peripheral blood mononuclear cell isolation

Human peripheral blood was collected and diluted with an equal volume of phosphate-buffered saline (PBS). The diluted blood was gently layered onto lymphocyte separation medium (TBD, Tianjin, China). Density gradient centrifugation was then performed at 2,000 rpm for 30 minutes at 20 °C without braking. After washing twice with PBS, the PBMCs were resuspended in RPMI 1640 medium (Gibco, Thermo Fisher Scientific, Waltham, MA, USA) for subsequent analysis.

### 2.5 Western blotting assay

Total protein was extracted from PBMCs using radio immunoprecipitation assay (RIPA) lysis buffer (Beyotime, Shanghai, China) supplemented with a protease and phosphatase inhibitor cocktail (Epizyme, Shanghai, China). Following a 30-minute incubation on ice, the lysates were centrifuged at 12,000 rpm for 5 minutes at 4 °C to remove cell debris. The supernatant was collected, and protein concentration was quantified using a bicinchoninic acid (BCA) assay kit (Beyotime, Shanghai, China). Samples were mixed with 5× loading buffer and stored at −80 °C until use. Equal amounts of protein were loaded onto 10% SDS-polyacrylamide gels, and electrophoresis was conducted at a constant voltage of 150 V. Proteins were transferred to a 0.45 µm PVDF membrane at a constant current of 400 mA for 30 minutes. Following transfer, the membranes were blocked with 5% (w/v) non-fat milk for 1 hour at room temperature. Subsequently, the membranes were incubated overnight at 4 °C with primary antibodies against STXBP3 (Cusabio, Wuhan, China, catlog: CSB-PA022907ESR2HU, epitop:343-592AA) and GAPDH (Servicebio, Wuhan, China). The membranes were then incubated for 1 hour at room temperature with horseradish peroxidase (HRP)-conjugated goat anti-rabbit IgG. Protein bands were visualized using enhanced chemiluminescence (ECL) reagents according to the manufacturer’s protocol (Epizyme, Shanghai, China). Band intensity was quantified using ImageJ software, and target protein expression was normalized to GAPDH levels.

### 2.6 Co-immunoprecipitation assay

HEK 293T cells were seeded in 6-well plates and transfected at approximately 80% confluence with the indicated plasmids using lipo8000 (Beyotime, Shanghai, China) according to the manufacturer’s instructions. The STXBP3-WT and STXBP3-mutant constructs were cloned into the pcDNA3.1 vector with a Flag tag fused to the c-terminus. A total of 2.5ug plasmid DNA and 4ul transfection reagent were used per well. Cells were harvested 48 h after transfection and then harvested in lysis buffer (Beyotime, China) containing protease inhibitors. Lysates were centrifuged at 12,000 rpm for 5 min at 4 °C to remove cell debris. After taking input samples, anti-Flag Magnetic Beads (Beyotime, China) were washed in Tris Buffered Saline (TBS) according to the manufacturer’s instructions, and added into cell lysates and incubated overnight at 4°C. After incubation, beads were washed three times with loading buffer (Epizyme, China) and boiled at 95°C for 5 min.

Co-immunoprecipitation experiments were performed in four independent experiments. Band intensities were quantified using ImageJ. To quantify the relative STXBP3–STX4 interaction, the co-immunoprecipitated STX4 signal was normalized to the corresponding immunoprecipitated STXBP3 signal (IP-STX4/IP-STXBP3). And the co-immunoprecipitated STX4 signal was also normalized to the corresponding input STXBP3 signal (IP-STX4/Input-STXBP3). For each normalization approach, the resulting ratios were directly compared between the WT and mutant groups. Statistical comparisons were performed using t-test.

### 2.7 Reverse transcription-polymerase chain reaction

Total RNA was extracted from PBMCs using RNAiso Plus (Takara, Dalian, China) following the manufacturer’s instructions. The concentration and quality of the isolated RNA were assessed using a UV5Nano spectrophotometer (Mettler Toledo, Greifensee, Switzerland) and 1% agarose gel electrophoresis, respectively. Subsequently, 1 μg of total RNA was reverse-transcribed into cDNA using the Evo M-MLV RT Mix Kit (Accurate Biology, Changsha, China). The specific primers used were as follows: Forward, 5’-TTCAGGCAATGGCATATGAT-3’ and Reverse, 5’-CTGAACCATTCCATACTGCT-3’.

### 2.8 RNA sequencing

cDNA libraries were constructed from all samples using the VAHTS Universal V10 RNA-seq Library Prep Kit (Premixed Version) (Vazyme, China), followed by sequencing on an Illumina novaXplus. Sequencing reads were aligned to the GRCh37 human reference genome using HISAT2 (v2.1.0), and transcript quantification was subsequently performed. Differential alternative splicing events were analyzed using rMATS (v4.1.2), and the results were visualized by rmats2sashimiplot (v2.0.4). Significant differential splicing events were defined as those with an FDR < 0.05 and an absolute inclusion level difference |IncLevelDifference| > 0.01.

### 2.9 Literature review

A systematic literature search of the published literature in PubMed database was performed using the following search strategy: (“STXBP3”[Title/Abstract] OR “syntaxin-binding protein 3”[Title/Abstract] OR “syntaxin binding protein 3”[Title/Abstract] OR “Munc18c”[Title/Abstract] OR “Munc18-3”[Title/Abstract] OR “Munc18 3”[Title/Abstract]), following the Preferred Reporting Items for Systematic Reviews and Meta-Analyses (PRISMA) guidelines (**Figure 1**). The final search was conducted on 22 July 2026.

**Figure 1:**
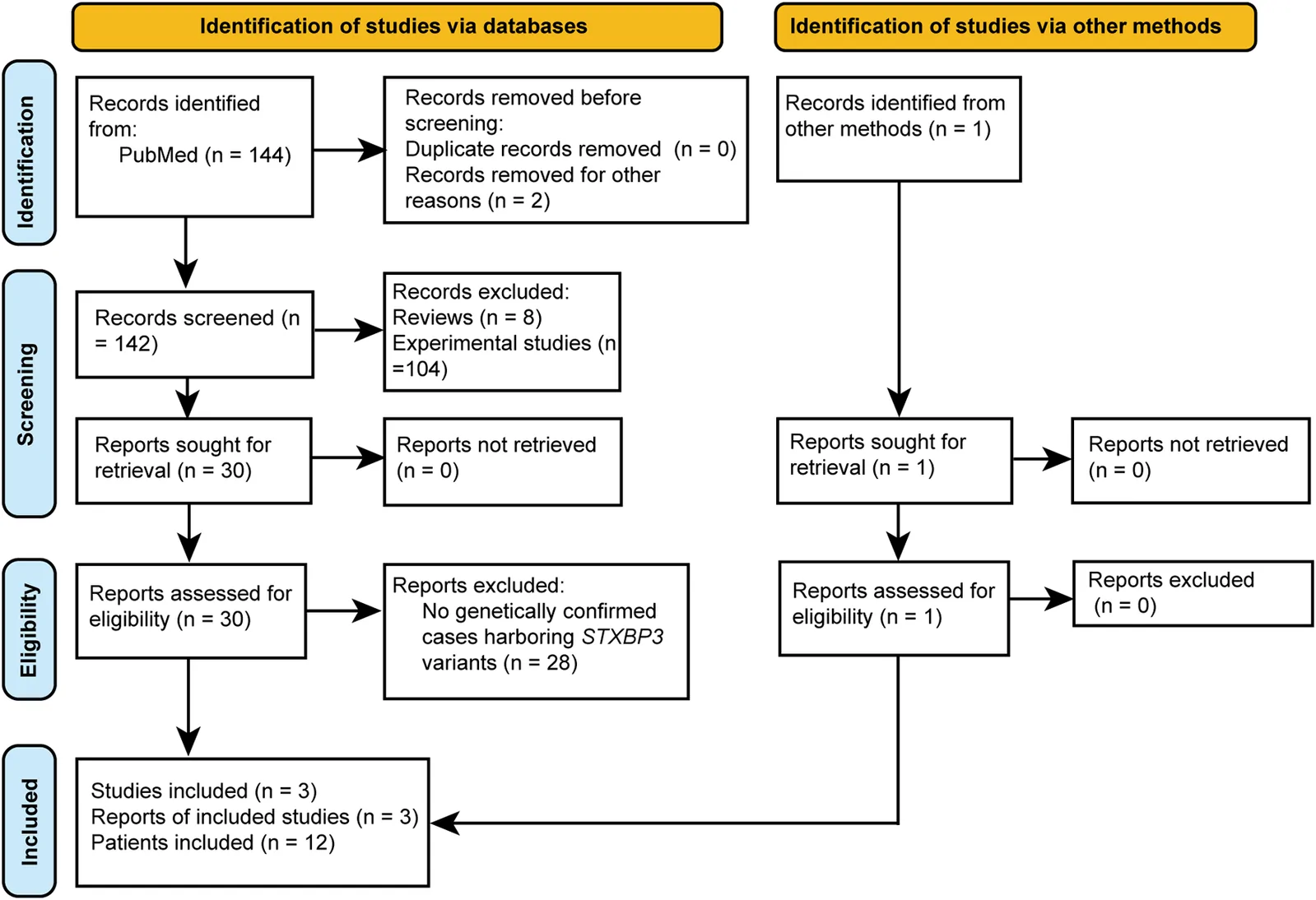
The PRISMA flow diagram.

After duplicate and retracted articles were removed, records were screened by title and abstract, followed by full-text eligibility assessment. Only articles containing individual clinical data were included. For patients reported in more than one publication, duplicated cases were counted only once in final analysis.

## 3 Results

### 3.1 Clinical features of the patient

The proband was a Chinese male infant from non-consanguineous parents. The father had a history of tricuspid valve malformation, and the mother had a history of hypothyroidism. The proband’s dizygotic twin sister was healthy, and there was no family history of gastrointestinal diseases (**Figure 2A**). Neither parent carried *STXBP3* variant, confirming its *de novo* occurrence in the proband. Therefore, the tricuspid valve malformation in the father and hypothyroidism in the mother were considered unlikely to be related to the *STXBP3* variant.

**Figure 2:**
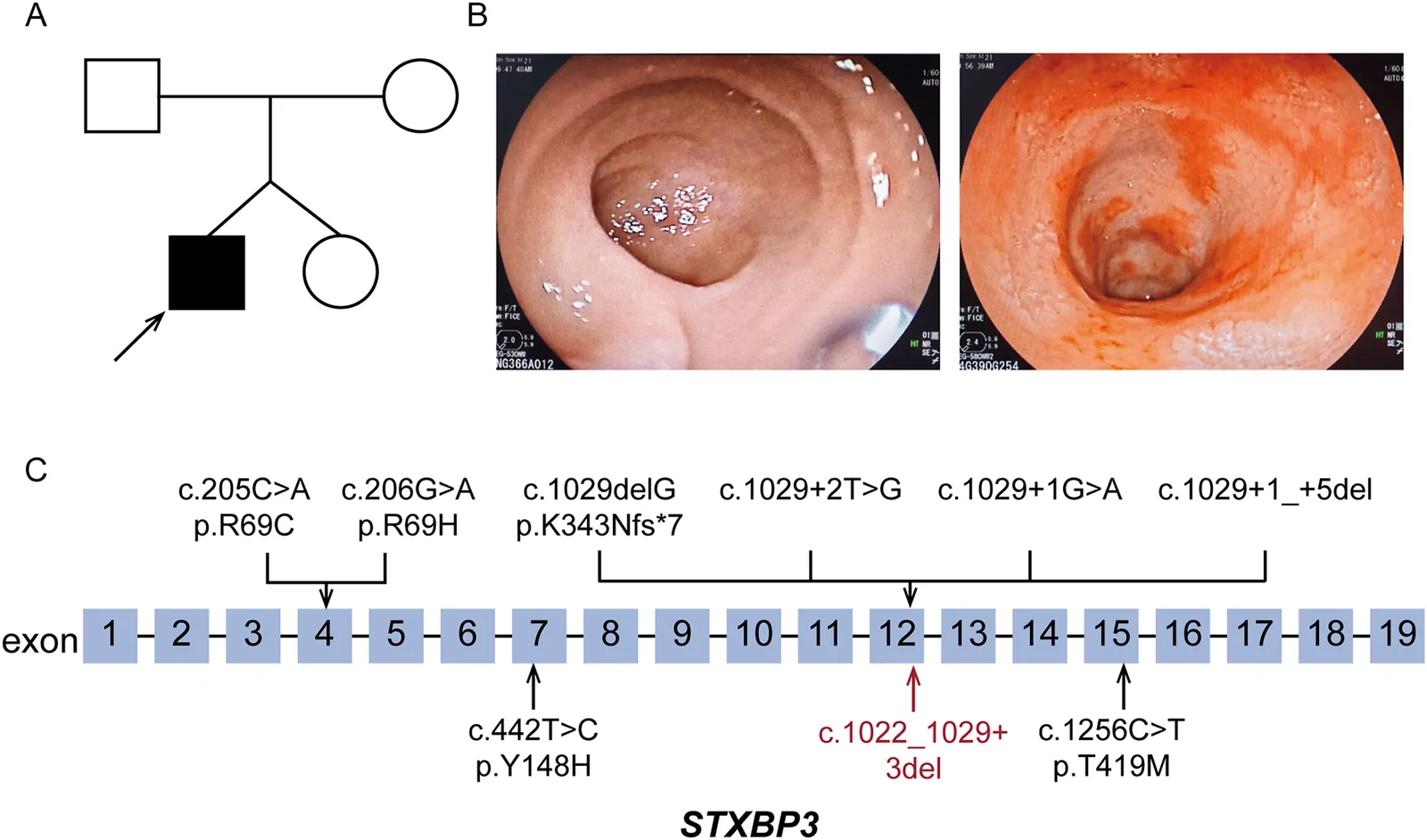
Clinical features of the proband. **(A)** Pedigree chart of the proband. **(B)** Representative images of gastrointestinal endoscopy. **(C)** Schematic diagram showing reported *STXBP3* variants. The variant identified in this case is highlighted in red.

At 20 days of age, the proband developed diarrhea with blood and mucus in stool. The diarrhea frequency was 10–20 times per day. Due to persistent diarrhea and recurrent infection, he was referred to our center for further evaluation and management. At 41 days of age, endoscopic examination revealed proctocolitis with multiple ulcers in the colon and rectum; the largest ulcer measured 0.3–0.5 cm in diameter (**Figure 2B**). Histopathological examination of the colorectal mucosa revealed variable crypt architectural distortion, mild focal active inflammation with occasional crypt abscesses, prominent eosinophilic infiltration (>100 cells/HPF), and immunohistochemical staining showed markedly reduced mucosal T cells, CD20+ B cells and CD138+ plasma cells, with only occasional IgA+ plasma cells and no detectable IgG+ or IgM+ plasma cells.

Given his recurrent infections, comprehensive immunological assessments were performed when he was 1 month old, which revealed hypogammaglobulinemia and reduced numbers of CD8^+^ T cells and NK cells (**Table 1**). Further testing, including a normal respiratory burst assay and detailed lymphocyte subpopulation analysis, demonstrated preserved naïve T-cell counts, thereby effectively ruling out severe combined immunodeficiency (SCID) (**Table 2**).

**Table 1:** Immunological profile of the patient at 1 month of age.

Parameter	Result	Reference range	Interpretation
CD16+CD56+ %	5.29	11−23%	Low
CD16+CD56+ (×10^6^/ml)	92.45	280−630	Low
CD3+ %	82.59	64−73%	High
CD3+ (×10^6^/ml)	1443.9	1410−3380	Normal
CD19+ %	10.62	14−21%	Low
CD19+ (×10^6^/ml)	185.72	45−820	Normal
CD4+ %	64.58	29−36%	High
CD4+ (×10^6^/ml)	1129.14	710−1840	Normal
CD8+ %	16.76	24−34%	Low
CD8+ (×10^6/^ml)	292.97	540−1360	Low
CD45+ (×10^6^/ml)	1748.351		
CD4+/CD8+	3.85	0.94−1.86	High
Complement C3 (g/L)	0.48	0.67−1.76	Low
Complement C4 (g/L)	0.15	0.1−0.4	Normal
CH50 (U/mL)	26	23−46	Normal
IgM (g/L)	0.11	0.1−0.7	Normal
IgA (g/L)	0.03	0.05−0.6	Low
IgG (g/L)	0.7	2.75−7.5	Low
IgE (IU/mL)	4.87	0−100	Normal

The percentages of CD3^+^, CD4^+^, and CD19^+^ cells were outside their respective reference ranges, whereas the absolute cell counts remained within the reference ranges, indicating altered relative lymphocyte subset distribution rather than abnormal absolute cell numbers.

**Table 2:** Detailed lymphocyte subpopulation analysis of the patient at 1 month of age.

Lymphocyte subset	Immunophenotype	Parent population	Result (%)	Reference range (%)	Interpretation
Total T cells	CD3+	Lymphocytes	81.05	54.28–71.67	High
CD4+ T cells	CD3+CD4+	Lymphocytes	55.8	33.72–52.43	High
CD8+ T cells	CD3+CD8+	Lymphocytes	22.47	14.08–24.70	Normal
Double-negative T cells	CD3+CD4−CD8−	CD3+ T cells	3.18	NA	
TCRαβ+ double-negative T cells	CD3+CD4−CD8−TCRαβ+	CD3+ T cells	0.44	0.33–1.12	Normal
TCRγδ+ T cells	CD3+TCRγδ+	Lymphocytes	2.69	3.32–7.40	Low
Naïve CD4+ T cells	CD3+CD4+CD27+CD45RA+	CD4+ T cells	94.05	69.15–88.10	High
CD4+ central memory T cells	CD3+CD4+CD27+CD45RA−	CD4+ T cells	5.75	10.11–28.20	Low
CD4+ effector memory T cells	CD3+CD4+CD27−CD45RA−	CD4+ T cells	0.11	0.28–2.10	Low
CD4+ terminally differentiated effector memory T cells	CD3+CD4+CD27−CD45RA+	CD4+ T cells	0.08	0.00–1.64	Normal
Naïve CD8+ T cells	CD3+CD8+CD27+CD45RA+	CD8+ T cells	96.45	68.90–94.60	High
CD8+ central memory T cells	CD3+CD8+CD27+CD45RA−	CD8+ T cells	3.46	5.14–25.55	Low
CD8+ effector memory T cells	CD3+CD8+CD27−CD45RA−	CD8+ T cells	0.03	0.10–4.95	Low
CD8+ terminally differentiated effector memory T cells	CD3+CD8+CD27−CD45RA+	CD8+ T cells	0.06	0.02–9.61	Normal
CD19+ B cells	CD19+	Lymphocytes	12.86	17.34–36.03	Low
Naïve B cells	CD19+CD27−IgD+	CD19+ B cells	96.16	87.55–94.85	High
Marginal zone B cells	CD19+CD27+IgD+	CD19+ B cells	2.93	1.70–6.50	Normal
Memory B cells	CD19+CD27+	CD19+ B cells	0.12	0.99–4.71	Low
Transitional B cells	CD19+CD38^high^CD24^high^	CD19+ B cells	20.43	15.05–29.95	Normal
Plasmablasts	CD19+CD38^high^	CD19+ B cells	0.02	0.60–3.95	Low

After discharge, the patient was initiated on oral mesalazine for gastrointestinal symptoms alongside regular intravenous immunoglobulin (IVIG) replacement therapy. At five months of age, mesalazine was discontinued due to lack of clinical response, while regular IVIG replacement therapy was maintained. Owing to persistent symptoms, a repeat endoscopy was conducted at 6 months of age, which revealed ongoing mucosal inflammation; pathological findings were consistent with VEOIBD associated with immunodeficiency. Thalidomide therapy was initiated but was withdrawn 6 months later after a follow-up endoscopy confirmed treatment failure. Given his immune disorder and refractory intestinal inflammation, prednisone was started at 14 months of age and led to partial symptomatic improvement, although clinical remission was not achieved. At 16 months, following a corticosteroid taper, the patient transitioned to the JAK inhibitor tofacitinib. However, tofacitinib was discontinued due to lack of efficacy. At 19 months of age, vedolizumab was introduced at dose of 90 mg per infusion (225mg/m^2^), administrated at approximately week 0, 2, 8,17. Before the fourth dose, therapeutic drug monitoring (TDM) revealed a low concentration of only 1.3 μg/mL, with no detectable anti-drug antibodies. Owing to family-related considerations, and the inadequate therapeutic response, the parents declined further vedolizumab treatment; therefore, further treatment optimization was not attempted. Currently, the patient is maintained solely on IVIG replacement therapy every 1.5 months. Despite this, his condition remains poorly controlled, with 5–10 stools per day and persistent anemia. Notably, longitudinal immunological evaluation showed that that the absolute NK cell, CD8+ T cell counts were reduced at several early assessments but subsequently fluctuated during follow-up, intermittently falling below and returning to the reference ranges. In contrast, absolute CD19+ B-cell counts were generally within the reference range throughout follow-up (**Supplementary Figure 1**). While longitudinal immunoglobulin measurements showed that, under regular IVIG replacement therapy, serum IgG levels were generally maintained around the lower limit of the age-adjusted reference range. Serum IgA and IgM levels gradually increased during follow-up and were largely within the age-adjusted reference ranges at later assessments. In contrast, serum IgE levels while have remained significantly elevated (**Supplementary Figure 2**).

Throughout the clinical course, the patient experienced recurrent infections, predominantly gastrointestinal infections presenting with vomiting and diarrhea. Documented infections included multiple episodes of rotavirus infection, amoebiasis, and oral candidiasis occurring in the absence of systemic corticosteroid therapy. Most infectious episodes improved with supportive and/or pathogen-directed treatment. He also developed episodes of severe diarrhea complicated by hyponatremia and hypoalbuminemia, eventually requiring admission to the ICU. Beyond gastrointestinal manifestations, the proband failed the newborn hearing screening and was diagnosed with sensorineural hearing loss at 6 months. Since the age of 2 years, he has frequently developed spontaneous ecchymoses that resolved slowly, although repeated coagulation studies were consistently normal.

### 3.2 Identification of a variant of *STXBP3* in the proband

WES identified a *de novo* heterozygous *STXBP3* variant (NM_007269.4: c.1022_1029 + 3del) in the proband (**Figure 2C**). This variant was absent from HGMD and gnomAD (allele frequency: 0.000%) and was classified as pathogenic according to ACMG guidelines. RNA-seq revealed reduced exon 12 inclusion in the patient (IncLevel = 0.36), consistent with increased exon 12 skipping, whereas the father showed complete exon 12 inclusion (IncLevel = 1.00) (**Figure 3A**). RT-PCR further demonstrated an aberrantly shorter transcript in the patient (**Figure 3B**), and Sanger sequencing confirmed exon 12 skipping in the abnormal transcript (**Figure 3C**).

**Figure 3:**
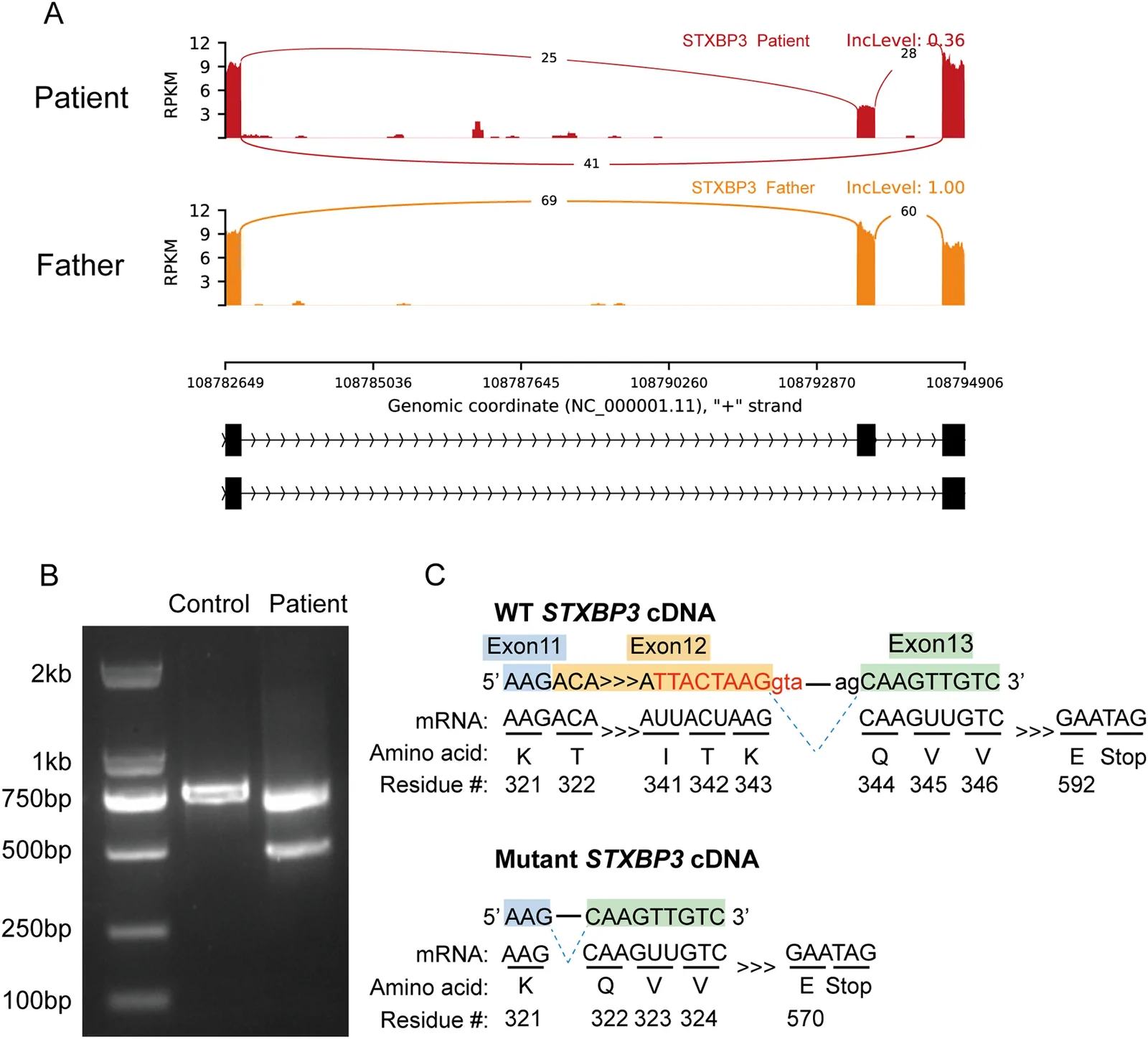
Aberrant splicing events in *STXBP3*. **(A)** Sashimi plot showing the aberrant splicing event in the patient. **(B)** Agarose gel electrophoresis results of the RT-PCR products. **(C)** Sanger sequencing of the RT-PCR product showed skipping of exon12 in patient’s transcription. The deletion bases are showed in red color. Exonic regions are represented by uppercase letters, while intronic regions are denoted by lowercase letters.

### 3.3 Aberrant splicing of *STXBP3* produced truncated protein while preserving STX4 binding

To elucidate the impact of the *STXBP3* variant on protein structure, we first evaluated STXBP3 protein expression in the patient sample. Western blot analysis revealed a reduction in overall STXBP3 levels and presence of a lower molecular weight band compared to healthy controls, consistent with a truncated STXBP3 isoform (**Figure 4A**). Notably, despite the patient harboring a heterozygous variant, only a single STXBP3 band was detected in PBMC lysates. Because the predicted molecular-weight difference between wild-type STXBP3 and the exon 12-skipped protein is only approximately 2.4 kDa, the two protein products may co-migrate and may not be distinguishable under the present experimental conditions. Densitometric analysis showed a modest trend toward lower STXBP3 expression in the patient PBMCs, but the difference was not statistically significant (**Figure 4B**). RNA-seq demonstrated the presence of both WT and exon 12-skipped transcripts, with approximately 25 and 41 supporting reads, respectively (**Figure 3A**).

**Figure 4:**
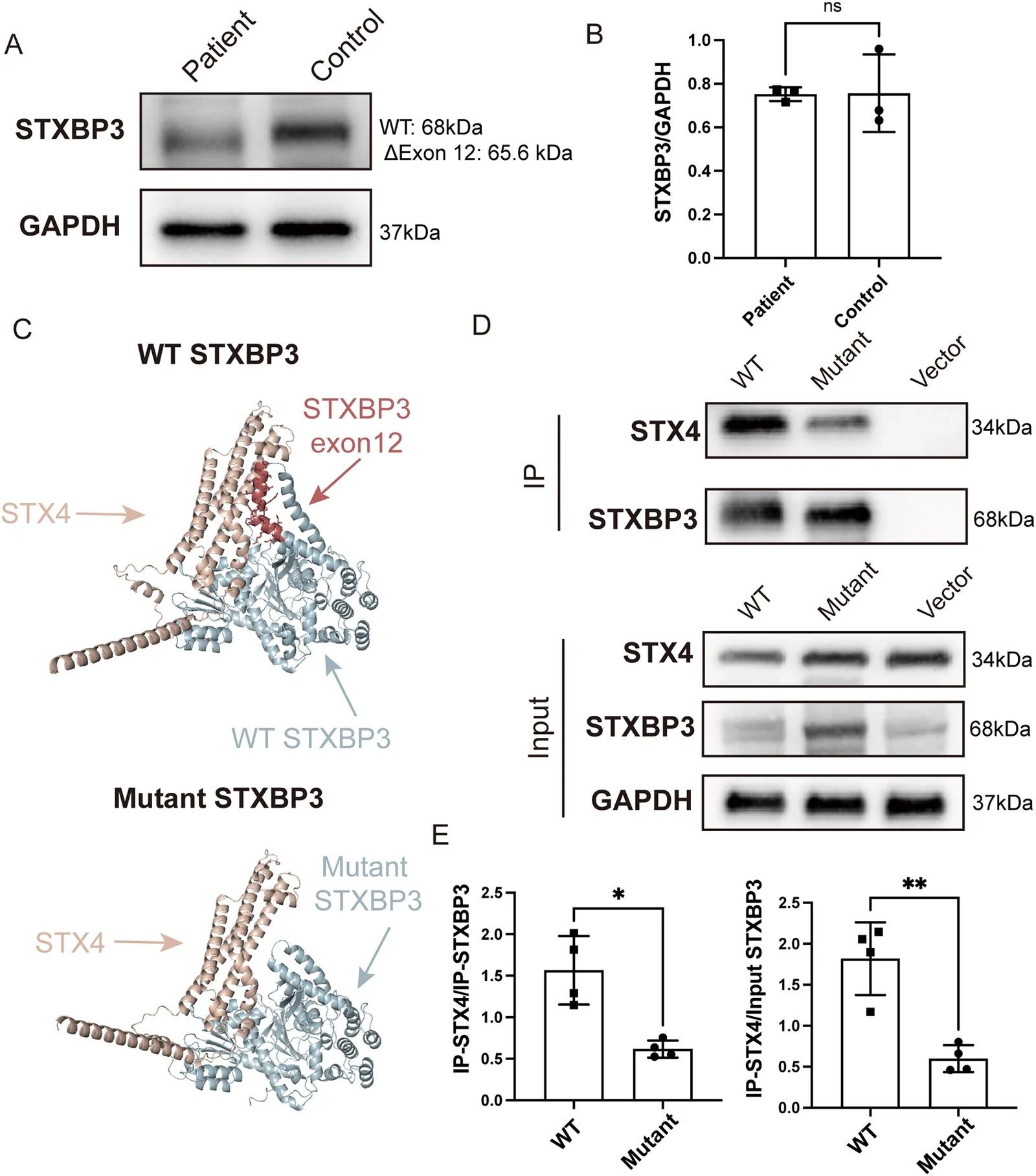
Exon skipping in *STXBP3* causes an internal deletion in the encoded protein. **(A)** Western blot analysis of the patient’s PBMCs. **(B)** Densitometric quantification of STXBP3 expression normalized to GAPDH from three independent experiments. Data are presented as mean ± SD. Statistical significance was assessed using t-test. **(C)** Alphafold3 predicted the binding between STXBP3 and STX4. Light blue represented the STXBP3 (left: mutant, right: WT), and the chain in pink represented the STX4 protein. The aminos residues that are missing in the c.1022_1029 + 3del are in red. **(D)** Representative co-IP assay showed remained binding between STX4 and mutant STXBP3. **(E)** Densitometric quantification from four independent experiments of co-IP assay. *p < 0.05, **p<0.01.

To assess the functional changes of mutant STXBP3, AlphaFold3 was used to model the WT and mutant complexes with STX4 (**Figure 4C**). The top-ranked WT and mutant models showed comparable global confidence metrics, with interface predicted TM scores (ipTM) of 0.75 and 0.76, predicted TM scores (pTM) of 0.81 and 0.81, and mean bidirectional inter-chain predicted aligned error (PAE) values of 16.18 Å and 16.50 Å, respectively. Nevertheless, residues 322–343 are located within the predicted STX4-contacting interface. Deletion of this segment could remove specific local contacts or alter the shape of the binding surface without markedly affecting the global confidence metrics. Co-IP assay confirmed that the interaction between STXBP3 and STX4 retained, but was impaired significantly (**Figures 4D, E**). Together, these findings suggest that the variant remodels the binding interface rather than completely disrupting STX4 association. Together, the structural prediction and biochemical findings suggest that the variant may locally remodel the binding interface and weaken, rather than completely abolish, STX4 association.

### 3.4 Literature review of reported cases

We systematically reviewed the literature regarding the clinical features and treatment strategies of patients with *STXBP3* gene variants reported to date (**Table 3**) (18, 23, 24). A total of 12 patients has been previously reported globally, involving eight distinct *STXBP3* variants. Including the patient in the current study, the total cohort comprised 13 individuals (nine males and four females) carrying 18 mutant alleles. Regarding variant types, missense variants accounted for 44.4% (8/18 alleles), while splice-site variants accounted for 38.9% (7/18 alleles). All reported splice-site and deletion variants followed an autosomal dominant (AD) inheritance pattern, whereas missense variants followed an autosomal recessive (AR) pattern.

**Table 3:** Clinical and genetic characteristics of patients with *STXBP3* variants.

Characteristics	P1	P2	P3	P4	P5	P6	P7	P8	P9	P10	P11	P12	P13
cDNA	c.1029delG	c.1029 + 1G>A	c.1029 + 1G>A	c.1029 + 1G>A	c.1029 + 1G>A	c.205C>T c.1256C>T	c.205C>T c.1256C>T	c.205C>T c.1256C>T	c.206G>A c.442T>C	c.206G>A c.442T>C	c.1029 + 1_1029 + 5del	c.1029 + 2T>G	c.1022_1029 + 3del
Protein	p.K343Nfs*7	NA	NA	NA	NA	p.R69C p.T419M	p.R69C p.T419M	p.R69C p.T419M	p.R69H p.Y148H	p.R69H p.Y148H	NA	NA	NA
Variant type	frameshift	splice site	splice site	splice site	splice site	missense	missense	missense	missense	missense	splice site	splice site	splice site
Inheritance pattern	AD	AD	AD	AD	AD	AR	AR	AR	AR	AR	AD	AD	AD
Zygosity	Het	Het	Het	Het	Het	Compound het	Compound het	Compound het	Compound het	Compound het	Het	Het	Het
Exon	exon12	intron12	intron12	intron12	intron12	exon4 exon15	exon4 exon15	exon4 exon15	exon4 exon7	exon4 exon7	intron12	intron12	exon12, intron12
ACMG	P	P	P	P	P	LP	LP	LP	LP	LP	P	P	P
Segregation	*De novo*	*De novo*	Paternal	Paternal	Unknown	Paternal/Maternal	Paternal/Maternal	Paternal/Maternal	Maternal	Maternal	*De novo*	NA	*De novo*
Sex	Male	Male	Male	Female	Male	Male	Female	Female	Female	Male	Male	Male	Male
GI involvement	IBD-U	Yes	Yes	Yes	Yes	secretory diarrhea	secretory diarrhea	secretory diarrhea	autoimmune enteropathy	autoimmune enteropathy	CD	IBD-U	UC
Adenomas	No	Yes	NA	NA	NA	NA	NA	NA	NA	NA	Yes	NA	No
Severe Infections	Yes	No	Yes	Yes	Yes	Yes	Yes	Yes	No	No	Yes	NA	Yes
NK cell numbers	L	L	N	NA	NA	NA	NA	NA	NA	N	NA	NA	L
CD8+ cell numbers	L	N	N	NA	NA	NA	NA	NA	NA	N	NA	NA	L
Hyponatraemia	Yes	Yes	Yes	Yes	NA	NA	NA	NA	No	No	Yes	NA	No
Hypoalbuminaemia	Yes	Yes	Yes	Yes	NA	Yes	Yes	Yes	Yes	Yes	Yes	NA	No
Congenital malformation	No	No	NA	NA	NA	Yes	Yes	Yes	Yes	Yes	No	NA	Yes
Hearing Loss	Yes	Yes	Yes	Yes	Yes	Yes	Yes	Yes	No	No	Yes	NA	Yes
TPN	Yes	Yes	Yes	NA	NA	Yes	Yes	Yes	Yes	Yes	NA	NA	No
Immunomodulator	Yes	Yes	NA	NA	NA	NA	NA	NA	Yes	Yes	Yes	NA	Yes
GI surgery	Yes	Yes	Yes	Yes	Yes	NA	NA	NA	NA	Yes	Yes	No	No
HSCT	Yes	No	No	No	No	No	No	No	No	No	No	Yes	No
IVIG	No	No	No	No	No	No	No	No	No	No	No	NA	Yes

GI, gastrointestinal; CD, Crohn’s disease; UC, ulcerative colitis; IBD-U, unclassified inflammatory bowel disease; TPN, total parenteral nutrition; HSCT, hematopoietic stem cell transplantation; IVIG, intravenous immunoglobulin; AD, autosomal dominant; AR, autosomal recessive; P, pathogenic; LP, likely pathogenic; L, low; N, normal; NA, not reported or data unavailable.

Clinically, all patients presented with diarrhea. Among those harboring missense variants, the predominant manifestations were secretory diarrhea or autoimmune enteropathy. Hypoalbuminemia was observed in 76.9% (10/13) of patients. Hyponatremia occurred in 38.5% (5/13) of cases, predominantly affecting the AD group (62.5%, 5/8) compared to 0/5 of the AR group. Immunological assessment revealed that 23.1% (3/13) of patients exhibited reduced counts of NK cells and/or CD8^+^ T cells, which appeared to occur more frequently in patients carrying splice-cite or deletion variants. Severe infections requiring hospitalization occurred in 61.5% (8/13) of patients. Tubular villous adenomas with dysplasia were identified in 15.4% (2/13) of cases. Regarding extraintestinal manifestations, sensorineural hearing loss was present in 76.9% (10/13) of patients; importantly, the absence of hearing impairment was observed exclusively in patients harboring missense variants. Congenital malformations were present in 46.2% (6/13) of the cohort, with marked differences between groups (**Table 4**). In the AD group, only one patient (12.5%, 1/8) presented with relatively mild anomalies, specifically hypospadias. In contrast, malformations were observed in 100% (5/5) of AR cases, involving complex features such as central hypotonia, joint contractures, syndactyly, and craniofacial abnormalities. Exploratory two-sided Fisher’s exact tests were performed for comparisons between the AD and AR groups (**Table 4**). Most clinical features did not differ significantly between the two groups. Congenital malformations showed a nominally significant difference between the AD and AR groups (12.5% vs. 100%, P = 0.048). However, this finding should be interpreted cautiously because of the small cohort size and multiple comparisons.

**Table 4:** Comparison of clinical features and treatments in AD and AR group.

Characteristics	AR (n=5)	AD (n=8)	Two-sided Fisher P
Adenomas	0.00% (n=0)	25.0% (n=2)	NA
Severe Infections	60.00%(n=3)	75% (n=6)	0.5227
Immune dysregulation	0.00% (n=0)	37.5% (n=3)	0.4000
Congenital malformation	100.00% (n=5)	12.5% (n=1)	0.0476
Hearing Loss	60.00%(n=3)	87.5% (n=7)	0.1515
TPN	100.00% (n=5)	37.5% (n=3)	0.4444
Immunomodulator	40.00% (n=2)	50.0% (n=4)	NA
GI surgery	20.00% (n=1)	75.0% (n=6)	1.0000
HSCT	0.00% (n=0)	25.0% (n=2)	0.4872
IVIG	0.00% (n=0)	12.5% (n=1)	1.0000

Data are presented as n (%), with percentages calculated using the total number of patients in each inheritance group as the denominator. Comparisons between AD and AR groups were performed using two-sided Fisher’s exact test based only on patients with available Yes/No data. Patients with unknown or unavailable information were excluded from the statistical analysis but retained in the denominator for percentage calculation. Statistical testing was not performed when one group had no available data or when there was no variation in the observed outcome. NA, not applicable; P < 0.05. GI, gastrointestinal; TPN, total parenteral nutrition; HSCT, hematopoietic stem cell transplantation; IVIG, intravenous immunoglobulin; AD, autosomal dominant; AR, autosomal recessive.

Regarding treatment strategies, gastrointestinal surgical intervention was performed in 53.8% (7/13) of patients, and total parenteral nutrition (TPN) support was required in 53.8% (7/13). Immunomodulators were administered to 46.2% (6/13) of patients, while vedolizumab was utilized in two cases. Notably, two patients underwent hematopoietic stem cell transplantation (HSCT), achieving complete resolution of gastrointestinal symptoms. Regarding prognosis, three patients dependent on TPN reached adulthood with clinical improvement; however, one patient died due to immune-related complications despite supportive care.

## 4 Discussion

*STXBP3* has been implicated as a genetic cause of VEOIBD. In this study, we describe a patient manifesting a complex phenotype characterized by VEOIBD, sensorineural hearing loss, immunodeficiency, and hypospadias, leading to the identification of a novel pathogenic *STXBP3* splice-site variant (c.1022_c.1029 + 3del). The results of RNA-seq and RT-PCR verified the existence of exon 12 skipping, which led to an internal deletion of STXBP3 protein. At the protein level, immunoblotting showed an altered STXBP3 band size rather than a marked reduction in overall protein abundance. Clinically, our patient showed a core phenotype similar to that reported in the AD *STXBP3* subgroup. However, the molecular findings suggest that the underlying pathogenic mechanism may not be entirely the same.

Our literature review suggests that a tentative genotype-phenotype correlation may exist in *STXBP3*-related disease. Splice-site and frameshift variants appeared to be more frequently reported in AD cases, which often presented with VEOIBD, sensorineural hearing loss, and immune dysregulation. Conversely, missense variants are more often reported in an AR inheritance pattern and tended to be associated with complex congenital malformations and hypotonia, while gastrointestinal involvement is also observed and hearing loss was reported in one of the two families. Given the limited number of reported cases and the incompleteness of some clinical data, these patterns should be interpreted cautiously, but they nevertheless suggest possible phenotypic differences according to variant type and inheritance pattern.

Previous studies have suggested haploinsufficiency as a likely mechanism for autosomal dominant *STXBP3*-related disease (18). In our case, STXBP3 protein expression in PBMCs from the patient showed a modest but non-significant reduction, suggesting that reduced protein dosage may contribute to disease pathogenesis but may not fully explain the phenotype (**Figure 4A**). However, our co-IP assay showed that the mutant STXBP3 protein retained detectable, but reduced, binding capacity with STX4 compared with WT STXBP3, indicating that the STXBP3-STX4 interaction was impaired but not completely abolished. These findings suggest that the pathogenic mechanism in our case may not be fully explained by haploinsufficiency alone, but may also involve additional effects related to the mutant protein. Specifically, because the mutant protein is expressed at near-WT levels and retains residual STX4 binding, it may compete with WT STXBP3 for STX4 while failing to support normal SNARE-complex turnover and vesicle fusion, thereby exerting a dominant-negative effect. Partial loss of function caused by impaired STX4 binding and altered phosphorylation-dependent regulation may also contribute. These mechanisms could be distinguished through analyses of SNARE-complex assembly and disassembly, vesicle-fusion activity, NK-cell granule release, and insulin-stimulated GLUT4 translocation.

This study also has important implications for clinical diagnosis and management. From a therapeutic perspective, patients in the autosomal dominant group often exhibit pronounced immunodeficiency and are prone to hyponatremia secondary to recurrent diarrhea, highlighting the need for close infection surveillance and timely supportive care. Notably, Case P1 and Case P12 achieved remission after hematopoietic stem cell transplantation (HSCT). Drawing parallels with *STXBP2*-associated IBD, in which patients often show only partial responses to conventional immunosuppressive therapy (25), HSCT can be a potential therapeutic option in selected severe cases, although complete control of colitis remains challenging. However, given the limited available evidence in *STXBP3*-related disease, the feasibility and timing of HSCT should be individualized according to disease severity, treatment response, infection burden, organ involvement, and overall clinical course. In the present patient, HSCT was not considered a priority treatment option at this stage, but may be reconsidered in the event of disease progression or clinical deterioration. Other therapeutic experiences further illustrate the heterogeneity of treatment responses within the AD group. Case P11 achieved remission of enteritis through combination therapy with methotrexate (MTX) and vedolizumab (VDZ), suggesting that immunomodulatory therapy together with VDZ may be effective in selected patients. However, our patient showed a refractory clinical course, with poor or absent responses to mesalazine, thalidomide, tofacitinib, and VDZ, and only partial improvement with prednisone and IVIG. In particular, the lack of response to tofacitinib does not support a clear therapeutic benefit of JAK inhibition in this patient. Given the role of STXBP3 in intracellular vesicle trafficking and secretion, the underlying disease mechanisms may involve pathways beyond JAK–STAT-mediated inflammation, although this possibility requires further investigation. At present, the patient remains on regular IVIG replacement therapy, while intestinal disease control remains unsatisfactory, with persistent diarrhea and anemia.

The immunological findings in this patient also warrant careful interpretation. The marked reduction of mucosal B cells and plasma cells, together with sparse or absent IgA+ plasma cells, suggests an abnormality of the mucosal B cell/plasma cell compartment. In contrast, the persistent hypogammaglobulinemia should be interpreted with caution. Serum IgG levels in early infancy are difficult to interpret in the setting of severe chronic diarrhea, because potential gastrointestinal immunoglobulin loss may influence. Moreover, specific antibody responses could not be assessed because immunoglobulin replacement therapy was initiated early in infancy. Thus, although the available findings raise the possibility of B cell dysfunction, definitive demonstration of an intrinsic functional B-cell defect would require evidence of impaired specific antibody responses. Given the severity of the patient’s clinical condition, IVIG replacement was nevertheless clinically justified. The recent occurrence of ecchymoses may also warrant further evaluation of platelet function, given the reported involvement of STXBP3 in platelet-related secretory processes.

By comparison, management of the AR group primarily relies on multidisciplinary support, including total parenteral nutrition (TPN) and neurorehabilitation. The use of immunomodulators in this group has yielded variable responses and carries significant risks. For instance, Case P9 developed severe complications culminating in liver failure. Overall, these observations underscore the marked clinical heterogeneity of *STXBP3*-related disease and highlight the need for individualized management strategies based on the predominant gastrointestinal, immunological, and systemic manifestations of each patient.

These findings should be interpreted in light of several limitations. As a single-case report, the generalizability of our findings is limited. Although the pathogenicity of the *STXBP3* variant was supported by genomic, transcriptomic, and protein-level analyses, the underlying mechanism remains incompletely understood. While abnormal STXBP3 protein expression was confirmed in the patient’s PBMCs, the functional assays were conducted in 293T cells, which may not fully reflect the effects of the variant in disease-relevant cell types. In addition, although our case broadens the clinical and immunologic spectrum of *STXBP3*-associated disease, further studies are required to clarify its relationship to the broader IEI spectrum.

In summary, the identification of the novel c.1022_1029 + 3del variant and the literature review expand our understanding of *STXBP3*-related disorders. Given the small number of reported cases and incomplete clinical data, the observed differences between the AD and AR subgroups should be regarded as a preliminary, hypothesis-generating framework rather than a definitive genotype–phenotype correlation. Although the present case shows broad clinical overlap with previously reported AD cases, our functional findings suggest that its pathogenic mechanism may not be explained solely by simple haploinsufficiency. Further studies involving larger cohorts and additional functional analyses are required to determine whether different classes of STXBP3 variants are associated with distinct phenotypic patterns or molecular mechanisms.

## Data Availability

The raw sequence data reported in this paper have been deposited in the Genome Sequence Archive (Genomics, Proteomics & Bioinformatics 2025) in National Genomics Data Center (Nucleic Acids Res 2025), China National Center for Bioinformation / Beijing Institute of Genomics, Chinese Academy of Sciences (GSA-Human: HRA031445) that are publicly accessible at https://ngdc.cncb.ac.cn/gsa-human.
