# Supplementary material for: Integrated genetic and functional analyses reveal the pathogenic effects of a novel STXBP3 variant in very-early-onset inflammatory bowel disease with immunodeficiency
Source: Front Immunol. 2026 Oct 5;17:1871633. doi: 10.3389/fimmu.2026.1871633 (PMC13677368; doi:10.3389/fimmu.2026.1871633)
Supplement: Supplementary file 1 [file SupplementaryFile1.docx]

Supplementary Material

# 1 Supplementary Figures and Tables

## 1.1 Supplementary Figures

**Supplementary Figure 1.** Longitudinal immune cells and immunoglobulin measurements. Serial measurements of (A) NK-cell count, (B) CD8^+^ T-cell count, (C) CD4^+^ T-cell count, (D) CD19^+^ T-cell count, (E)CD3^+^ T-cell count are shown. The gray shaded areas indicate the age-specific reference ranges applicable at each time point. Horizontal bars indicate continuous treatment periods, including IVIG, thalidomide, corticosteroids, and tofacitinib. Open circles indicate individual vedolizumab administrations. IVIG was initiated before the first immunological assessment at our center, and was continued throughout follow-up.

**Supplementary Figure 2.** Longitudinal immunoglobulin measurements. Serial measurements of (A) IgG, (B) IgA, (C) IgM, and (D) IgE are shown. The gray shaded areas indicate the age-specific reference ranges applicable at each time point. In panel D, the IgE concentration is displayed on a logarithmic scale, and the dashed horizontal line indicates 500 IU/mL. Horizontal bars indicate continuous treatment periods, including IVIG, thalidomide, corticosteroids, and tofacitinib. Open circles indicate individual vedolizumab administrations. IVIG was initiated before the first immunological assessment at our center, and was continued throughout follow-up.
